# Aberrant MCT4 and GLUT1 expression is correlated with early recurrence and poor prognosis of hepatocellular carcinoma after hepatectomy

**DOI:** 10.1002/cam4.1521

**Published:** 2018-10-10

**Authors:** Hai‐Long Chen, Han‐Yue OuYang, Yong Le, Peng Jiang, Hui Tang, Zi‐Shan Yu, Min‐Ke He, Yun‐Qiang Tang, Ming Shi

**Affiliations:** ^1^ Department of Hepatobiliary Oncology Sun Yat‐sen University Cancer Center Guangzhou 510060 Guangdong China; ^2^ Collaborative Innovation Center for Cancer Medicine Sun Yat‐sen University Cancer Center Guangzhou 510060 Guangdong China; ^3^ Sate Key Laboratory of Oncology in South Guangzhou 510060 Guangdong China; ^4^ GI Cell Biology Research Laboratory Children's Hospital Boston and Harvard Medical School Boston MA USA; ^5^ Fourth Internal Medicine Affiliated Cancer Hospital & Institute of Guangzhou Medical University Guangzhou 510095 Guangdong China; ^6^ Department of Hepatobiliary Oncology Affiliated Cancer Hospital & Institute of Guangzhou Medical University Guangzhou 510095 Guangdong China

**Keywords:** GLUT1, hepatocellular carcinoma, MCT4, prognosis

## Abstract

The tumor microenvironment is a key determinant of cancer cell biology. The microenvironment is a complex mixture of tumor cells, stromal cells, and proteins, extracellular matrix, oxygen tension, and pH levels surrounding the cells that regulate the tumor progress. This study identified the prognostic factors associated with hepatocellular carcinoma (HCC) and MCT4 and GLUT1 expression levels in HCC specimens. In this study, we analyzed MCT4 and GLUT1 expression levels in tissue samples from 213 patients with HCC by immunohistochemical analyses and in HCC tumor tissues and matched adjacent nonneoplastic tissues by quantitative real‐time PCR. We conducted a prognostic analysis of the overall survival (OS) and time to recurrence (TTR) using immunoreactivity and other common clinical and pathological parameters. All variables with prognostic impact were further analyzed by multivariate analysis. We found that MCT4 and GLUT1 expression levels were significantly higher in tumor tissues than in adjacent nontumor tissues, and they were positively correlated with tumor size. Survival analysis showed that patients with high expression levels of MCT4 or GLUT1 had a poor OS and TTR. In patients with HCC, MCT4 expression was an independent negative prognostic factor for OS (hazard ratio [HR] = 1.617; 95% confidence interval [CI] = 1.102–2.374; *P* = 0.014), and metabolic indicators were independent prognostic factors for OS (HR = 1.617, 95% CI = 1.102−2.374, *P* = 0.006) and TTR (HR = 1.348, 95% CI = 1.079−1.685, *P* = 0.009). Interestingly, patients with positive metabolic indicator expression in tumor cells had a significantly shorter OS and earlier TTR than those with negative metabolic indicator expression in tumor cells in the ≤5 cm and >5 cm subgroups. In summary, using the expression of MCT4 and GLUT1 and their metabolic parameters to determine the metabolic status of tumors is promising for predicting the prognosis of patients with HCC.

## Introduction

Hepatocellular carcinoma (HCC) is one of the most common malignant tumors in the world, and its morbidity and mortality increase each year 1. In recent years, many advances in medical technology have been made, but liver cancer treatment commonly comprises surgical resection combined with radiofrequency ablation, interventional embolization, and targeted molecular therapies 2, 3, 4. However, most patients miss their opportunity to receive radical treatment because they have already reached an advanced stage of liver cancer by their first visit 5, 6. Therefore, it is of great importance to further explore the pathogenesis of HCC and to determine highly selective therapeutic targets for HCC prognosis to facilitate clinical HCC studies and improve the efficacy of HCC treatment.

Like many other solid cancers, the role of the tumor cell microenvironment is considered to be key in HCC development 7. With the exception of the traditional oxidative oxidation of glucose to pyruvate via the mitochondrial tricarboxylic acid cycle, the energy required for cancer cells comes from the anaerobic glycolysis of glucose 8. In this process, glucose is converted to lactate by lactate dehydrogenase in cancer cells (Warburg effect) 9. The formation and regeneration of hepatocellular lesions, as well as neovascularization, are also closely linked to hepatocyte anaerobic glycolysis 10. Lactate efflux occurs through the lactate‐activated transcription factor hypoxia‐induced factor 1α (HIF1α), which induces glucose transporter 1 (GLUT1), LDH‐A, and monocarboxylate transporters 11, 12, particularly monocarboxylic acid transporter 4 (MCT4) 13. The maintenance of glycolysis requires continuous lactic acid excretion from the cells by a group of monocarboxylic acid transporters; in this process, MCT4 promotes the transport of short‐chain carbohydrates, such as pyruvate and lactate, to maintain intracellular pH levels and glycolysis 14. MCT4 is highly expressed in lung cancer, renal cell carcinoma 15, breast cancer 16, and pancreatic cancer 17, and high levels of MCT4 expression are associated with a high apoptotic index 18.

In this study, we evaluated the metabolic status of HCC tissues by assessing MCT4 and GLUT1 expression. We also analyzed the prognostic significance of the expression of these transporters in patients with HCC.

## Materials and Methods

### Patients and samples

This study was performed strictly in accordance with the Helsinki Declaration. The Sun Yat‐sen University Cancer Center Research Ethics Committee approved this study, and all patients provided informed consent. Tumor and matched adjacent nontumorous tissues were consecutively collected from 213 patients with HCC during curative resection from December 2002 to November 2010 at Sun Yat‐sen University Cancer Center (Guangzhou, China). Diagnostic criteria were used to assess the postoperative pathology and were based on the criteria of the European Association for the Study of the Liver (EASL) and the European Organisation for Research and Treatment of Cancer (EORTC). All patients had complete medical history data. The criteria for inclusion in the study were as follows: (1) no anticancer treatment or distant metastasis prior to surgery; (2) no concurrent autoimmune diseases, HIV, or syphilis; and (3) follow‐up data were available. Patients classified as Child–Pugh class B or C were excluded from our study, as well as patients who had radical liver cancer surgery. Detailed clinicopathological parameters are listed in Table 1.

**Table 1:** Clinical characteristics of patients with hepatocellular carcinoma

Clinicopathological variable	Median (range)	No. of patients	%
Age (year)	50 (16–77)		
Gender			
Female		31	14.6
Male		182	85.4
HBsAg			
Negative		31	14.6
Positive		182	85.4
Serum AFP (ng/mL)	172.7 (0–121000.0)		
Tumor size (cm)	6.4 (1.6–15.6)		
Tumor number			
Solitary		148	69.5
Multiple		65	30.5
Microvascular invasion			
No		172	80.8
Yes		41	19.2
Differentiation grade			
I		15	7.0
II		104	48.8
III		90	42.3
IV		4	1.9
BCLC stage			
0		4	1.9
A		167	78.4
B		30	14.1
C		12	5.6
TNM stage			
I		137	64.3
II		42	19.7
III		34	16.0
AST (μ/L)	51.9 (12.3–182.6)		
ALT (μ/L)	47.3 (5.6–168.4)		
Liver cirrhosis			
No		98	46.0
Yes		115	54.0

AFP, alpha‐fetoprotein; BCLC, Barcelona Clinic Liver Cancer; AST, aspartate transaminase; ALT, alanine transaminase.

A total of 66 pairs of resected HCC and adjacent nonneoplastic liver tissues were collected from patients who had undergone hepatectomies for the curative treatment of HCC at the Cancer Center of SYSU from 2012 to 2014. None of the patients received neoadjuvant therapies, such as radiotherapy or chemotherapy, before surgery. Informed consent was obtained from patients regarding the use of their liver specimens for research.

### Follow‐up

The average postoperative follow‐up time in our study was 60.74 months. The longest postoperative follow‐up time was 126 months. Our primary study endpoint was the last follow‐up without recurrence or death. Overall survival (OS) was defined as the time until postoperative death or the last follow‐up. Time to recurrence (TTR) was defined as the interval between surgery and recurrence or between surgery and the last follow‐up for patients without recurrence. Postoperative follow‐up included abdominal ultrasound examinations or computed tomography studies every 3 months, serum alpha‐fetoprotein (AFP) measurements every 1–3 months, and hepatic artery angiography, bone imaging, or chest CT examination if necessary. If cancer recovery is to be achieved, it is considered necessary to use various treatments, including repeated hepatectomy, transcatheter arterial embolization, percutaneous ablation, and radiotherapy 19.

### Immunohistochemistry

Resected surgical specimens were fixed in formalin and embedded in paraffin. Then, the specimens were cut into 4‐μm sections and placed onto glass slides. The sections were sequentially dehydrated with xylene, paraffin, and ethanol and then rehydrated with a graded series of alcohol. Next, the tissue slides were treated with fresh 0.3% hydrogen peroxide solution for 10 min to block the endogenous peroxidase and subjected to antigen repair in 0.01 mol/L sodium citrate buffer (pH 6.0) for 25 min using a microwave oven.

The sections were then incubated with anti‐MCT4 antibody (1:500 dilution; Santa Cruz, Cat. # sc‐376101, Delaware Ave, CA, USA) or anti‐GLUT1 antibody (1:500 dilution; Abcam, Cat. # ab652, Cambridge, U.K.) overnight at 4°C. Next, the tissue sections were incubated with HRP‐labeled anti‐mouse/rabbit secondary antibody (Dako, Cat. # K5007, Glostrup, Denmark) for 1 h at room temperature. The reaction product was visualized using a nonbiotin horseradish peroxidase assay system according to the manufacturer's protocol, and the brown color indicates positive staining. All sections were counterstained with Mayer's hematoxylin and fixed in nonaqueous fixative.

H‐score 20 was used for semiquantitative analysis of immunoreactivity of MCT4 and GLUT1. The score was obtained using the formula: 3 × percentage of strongly staining +2 × percentage of moderately staining +1 × percentage of weakly staining, giving a range of 0–300. Positive immunoreactivity was defined as H‐score>0. Score was independently obtained by two of different professional pathologists who were blinded to the clinical data.

### RNA isolation and quantitative real‐time PCR

Total RNA was isolated from tissues and cell lines using TRIzol reagent (Invitrogen Life Technologies, Carlsbad, CA, USA). RNA (2 μg) was reverse‐transcribed using a SuperScript^®^ III First‐Strand Synthesis System (Invitrogen Life Technologies) according to the manufacturer's instructions. For real‐time PCR assays, cDNA was subjected to PCR amplification using SYBR Green (Toyobo, Kita‐Ku, Osaka, Japan) and a Roche LightCycler 480 System. GAPDH was used as an internal control. The primers were as follows:

MCT4

Forward: GTCATCTCTCTGCCCCACAT
Reverse: AGCACGGTCAATGAGAACAA

GLUT1

Forward: TTATTGCCCAGGTGTTCGGC
Reverse: GTAGCAGGGCTGGGATGAAG

GAPDH

Forward: GGTATGACAACGAATTTGGC
Reverse: GAGCACAGGGTACTTTATTG

### Statistical analysis

IBM SPSS (version 22; IBM Corporation, Armonk, NY, USA) and GraphPad Prism (version 7; GraphPad Software, La Jolla, CA, USA) were used for statistical analyses. OS and TTR curves were generated according to the Kaplan–Meier method, and the differences between the curves were analyzed by log‐rank test. Using the Cox proportional hazards model, significant prognostic factors determined by univariate analysis were entered into the multivariate analysis. The relationship between MCT4/GLUT1 expression and various clinicopathological parameters was analyzed by chi‐square test or Fisher's exact test. Pearson correlation analysis was used to analyze the correlation between MCT4 and GLUT1 staining scores. The results were considered statistically significant at *P* < 0.05.

## Results

### GLUT1 and MCT4 expression in HCC tumors and nontumor tissues

**Figure 1:**
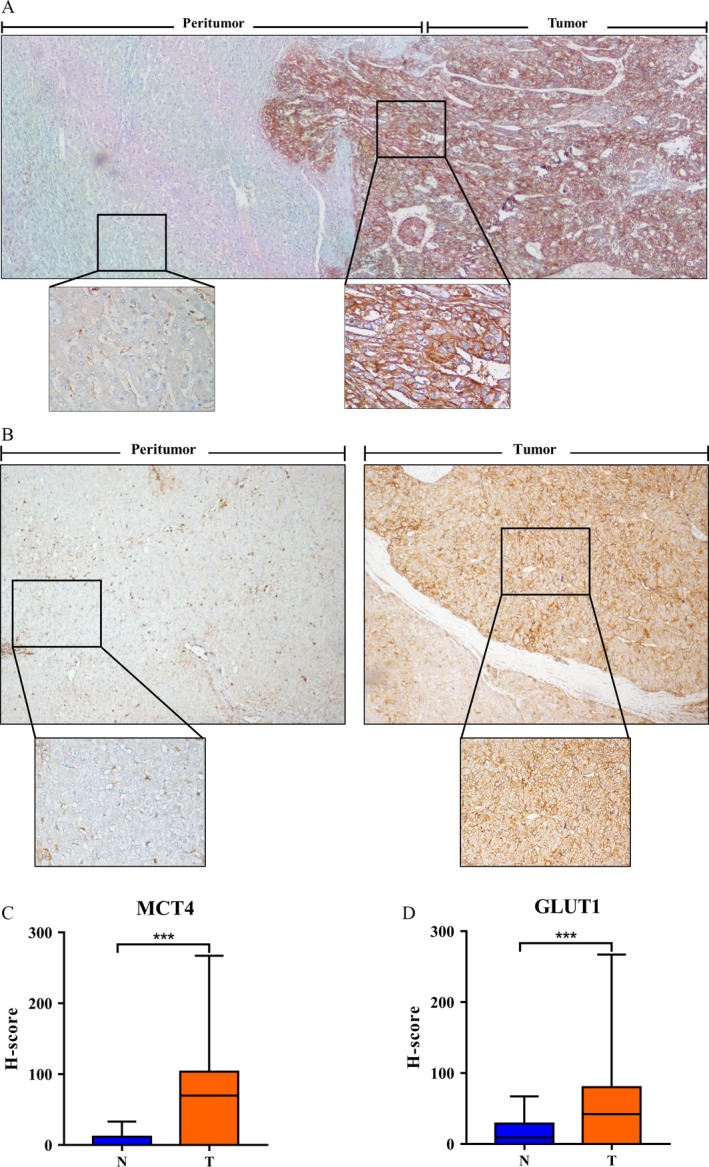
IHC characteristics of MCT4 and GLUT1 in HCC specimens. (A, B) IHC assays of MCT4 (A) and GLUT1 (B) expression in tumor (T) and adjacent nontumor tissues (N). (A) MCT4 expression levels in the tumor (T) tissues are significantly higher than those in the adjacent nontumor tissues (N) (40×). The bottom panel shows an enlargement of the indicated area (200×). (B) GLUT1 expression levels in the tumor (T) tissues are significantly higher than those in the adjacent nontumor tissues (N) (40×). The bottom panel shows an enlargement of the indicated area (200×). (C, D) MCT4 (C) and GLUT1 (D) expression levels in tumor (T) tissue are significantly higher than those in adjacent nontumor tissue (N). The IHC H‐scores are shown as mean with range (bars); *** indicates *P* < 0.01.

Immunohistochemical (IHC) staining was used to determine the expression of MCT4 and GLUT1 in 213 HCC samples (tumor and adjacent nontumor tissues). We found clear and distinguishable membrane staining for both MCT4 and GLUT1 in tumor tissue but weakened staining in the adjacent hepatocytes (Fig. 1A and B). MCT4 expression was significantly higher in tumor tissues (median score = 68.0) than in adjacent nontumor tissues (median score = 0.0; *P < *0.001; Fig. 1C). Similar results were found for GLUT1 (median score: tumor tissues [T] = 43.0, nontumor tissues [N] = 9.0; *P* < 0.001; Fig. 1D). These data indicate that GLUT1 expression and MCT4 expression are upregulated during tumorigenesis.

### MCT4/GLUT1 expression and its correlation with clinicopathological features

**Figure 2:**
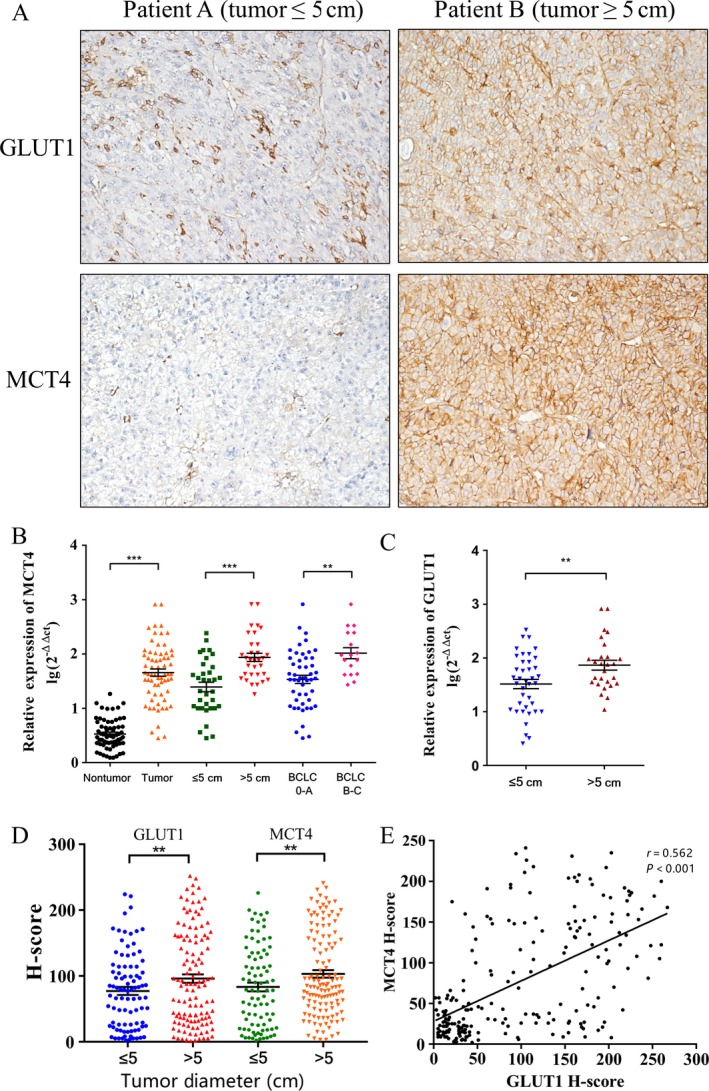
Association between the expression levels of GLUT1 and MCT4 with clinicopathological features. (A) IHC staining for GLUT1 and MCT4 in patients with tumor diameters less than or greater than 5 cm. (B, C) Quantitative real‐time PCR was used to determine the relative MCT4 expression in 66 pairs of HCC tumors and matched nontumor tissues, the correlation between tumor size as well as BCLC stage and MCT4 expression levels, and the correlation between tumor size and GLUT1 expression. Each point represents a sample. (B) MCT4 levels in HCC tissues were significantly higher than those in nontumor tissues (left two columns), and MCT4 was more highly expressed (middle two columns) in tumor samples larger than 5 cm in diameter. (C) Similar results were observed for GLUT1 in the same samples. (D) The IHC H‐scores for GLUT1 (left two columns) and MCT4 (right two columns) in patients with tumor diameter less or more than 5 cm. (E) GLUT1 expression level is positively correlated with MCT4 expression (*n* = 213). The IHC H‐scores are expressed as mean ± standard error of mean (bars); *** indicates *P* < 0.01, and ** indicates *P* < 0.05.

Table 1 summarizes the clinicopathological features of 213 patients with HCC. The median follow‐up time was 60.74 months (range, 1.6–126 months). During follow‐up, 118 (55.4%) patients died, and 104 (48.8%) patients were diagnosed with tumor recurrence. The median OS and TTR for patients were 61.6 and 22.2 months, respectively. Patients were divided into two groups according to the level of MCT4 and GLUT1 expression. The cutoff value of MCT4 and GLUT1 expression was 87.25 and 57.75, respectively, as determined by the receiver operating characteristic (ROC) curve. High expression levels of MCT4 and GLUT1 were present in 47.4% (101/213) and 59.2% (126/213) of all cases. To further evaluate the GLUT1/MCT4 expression patterns in HCC tumor tissues and adjacent nonneoplastic tissues, we also examined their levels in 66 pairs of matched HCC tumor and nontumor specimens by quantitative real‐time PCR. We found that the expression levels of MCT4 were remarkably higher in the HCC tumor tissues than in the matched adjacent nonneoplastic tissues (Fig. 2B). Moreover, the MCT4 mRNA levels in tumor tissues were significantly higher in patients with a tumor diameter >5 cm than in those with a tumor diameter ≤5 cm (*P* < 0.01; Fig. 2B). GLUT1 mRNA levels were also significantly higher in patients with bigger tumors than in those with smaller tumors (*P* < 0.05; Fig. 2C). Besides, the expression level of MCT4 mRNA was dissimilar in different Barcelona Clinic Liver Cancer (BCLC) stages of tumor tissue (*P* < 0.05; Fig. 2B).

We also analyzed the correlation between GLUT1/MCT4 expression status and clinicopathological features. MCT4 and GLUT1 expression levels correlated with tumor sizes (*P* < 0.05; Table 2). Patients with larger tumors tended to have higher MCT4 or GLUT1 expression levels (*P* < 0.01; Fig. 2A and D). In addition, MCT4 expression levels correlated with a poor BCLC stage (*P* = 0.002; Table 2). Furthermore, there was a positive correlation between MCT4 and GLUT1 expression (*r* = 0.562, *P* < 0.001; Fig. 2E).

**Table 2:** Correlation between MCT4/GLUT1 expression and clinicopathological parameters

Clinicopathological variable	NO.	MCT4 expression levels			GLUT1 expression levels		
		Low	High	*P* value	Low	High	*P* value
Age (years)							
≤50	103	57	46	0.435	42	61	0.984
>50	110	55	55		45	65	
Gender							
Female	31	16	15	0.907	13	18	0.894
Male	182	96	86		74	108	
HBsAg							
Negative	31	15	16	0.613	11	20	0.511
Positive	182	97	85		76	106	
Serum AFP (ng/mL)							
≤400	111	64	47	0.122	48	63	0.458
>400	102	48	54		39	63	
Tumor size (cm)							
≤5	90	38	52	**0.010**	30	60	**0.046**
>5	123	74	49		57	66	
Tumor number							
Solitary	148	77	71	0.807	60	88	0.891
Multiple	65	35	30		27	38	
Microvascular invasion							
No	172	93	79	0.257	74	98	0.185
Yes	41	18	23		13	28	
Differentiation grade							
I+II	119	62	57	0.874	49	70	0.912
III+IV	94	50	44		38	56	
BCLC stage							
0–A	171	99	72	**0.002**	75	96	0.071
B–C	42	13	29		12	30	
TNM stage							
I	137	68	69	0.247	55	82	0.780
II–IV	76	44	32		32	44	
Recurrence							
No	109	48	61	**0.011**	37	72	**0.036**
Yes	104	64	40		50	54	
AST (μ/L)							
≤40	112	59	53	0.976	47	65	0.726
>40	101	53	48		40	61	
ALT (μ/L)							
≤40	122	64	58	0.967	53	69	0.372
>40	91	48	43		34	57	
Liver cirrhosis							
No	98	49	49	0.486	38	60	0.571
Yes	115	63	115		49	66	

AFP, alpha‐fetoprotein; BCLC, Barcelona Clinic Liver Cancer; AST, aspartate transaminase; ALT, alanine transaminase.

Bold values (*P* < 0.05) are statistically significant.

### Prognostic value of MCT4 and GLUT1 expression in HCC

Univariate analysis of MCT4/GLUT1 status and routine clinicopathological parameters showed that GLUT1 overexpression, MCT4 overexpression, high alpha‐fetoprotein levels, large tumor size, multiple tumors, poor BCLC stage, poor tumor–node–metastasis (TNM) stage, and microvascular invasion are unfavorable predictors of OS in patients with HCC (Table 3). In addition, high GLUT1 expression levels, high MCT4 expression levels, multiple tumors, and poor TNM stage were significantly associated with shorter TTR in patients with HCC (Table 3).

**Table 3:** Univariate and multivariate analyses of prognostic factors in HCC

Variables	OS			TTR		
	Univariate *P* value	Hazard ratio (95% CI)	*P* value	Univariate *P* value	Hazard ratio (95% CI)	*P* value
Age (years)	0.795			0.301		
Gender	0.866			0.660		
HBsAg	0.277			0.934		
Serum AFP (ng/mL)	0.020		N.A.	0.061		
Tumor size (cm)	0.001	1.900 (1.122–3.215)	**0.017**	0.097		
Tumor number	0.000	1.657 (1.061–2.587)	**0.026**	0.028		N.A.
Microvascular invasion	0.001	2.037 (1.267–3.272)	**0.003**	0.000	2.508 (1.457–4.316)	**0.001**
Differentiation grade	0.569			0.406		
BCLC stage	0.007	1.547 (1.023–2.337)	**0.038**	0.892		
TNM stage	0.015		N.A.	0.011		N.A.
AST (μ/L)	0.815			0.929		
ALT (μ/L)	0.348			0.991		
Liver cirrhosis	0.372			0.287		
MCT4 expression	0.001	1.617 (1.102–2.374)	**0.014**	0.004	1.930 (1.091–3.414)	**0.024**
GLUT1 expression	0.020		N.A.	0.044		N.A.

OS, overall survival; TTR, the time to recurrence; HR, hazard ratio; CI, confidence interval; N.A., not applicable; MCT4, monocarboxylate transporter 4; GLUT1, glucose transporter 1.

Univariate analysis, Cox proportional hazards regression model. Multivariate analysis, Cox proportional hazards regression model. Variables were adopted by univariate analysis.

Bold values (*P* < 0.05) are statistically significant.

**Figure 3:**
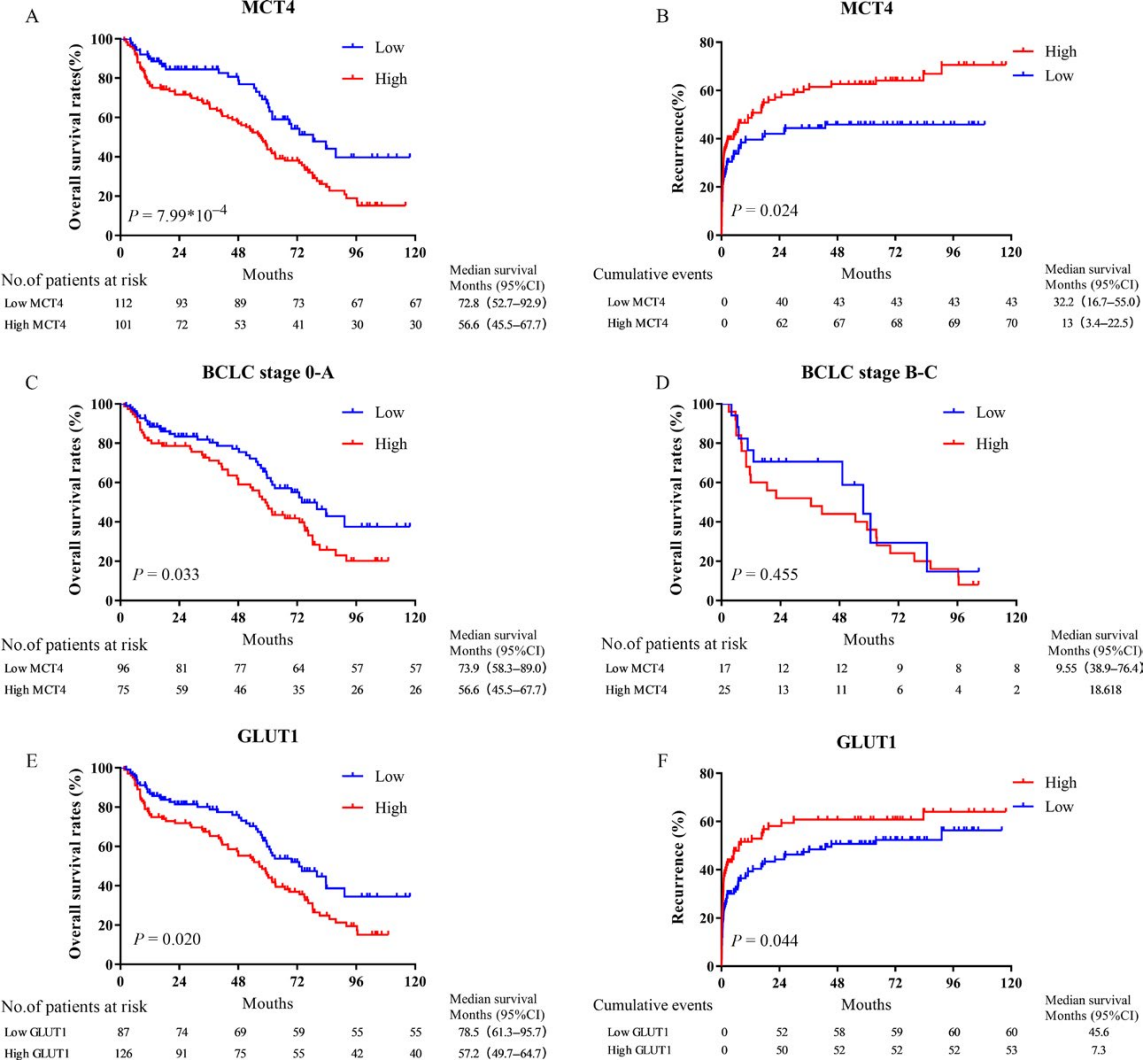
Prognostic significance of MCT4 expression and GLUT1 expression. (A, B) HCC patients with high MCT4 expression levels have a poor OS (A) and short TTR (B). (C, D) Kaplan–Meier analysis of the correlation between MCT4 expression in tumor cells and OS in BCLC stages 0–A (C) and stages B–C (D). (E, F) HCC patients with high GLUT1 expression levels have a poor OS (E) and short TTR (F).

Kaplan–Meier survival curves showed that patients with high GLUT1 expression levels had lower OS (*P* = 0.02; Fig. 3A) and higher TTR rates (*P* = 0.044; Fig. 3B). The 5‐year OS and TTR rates in the high‐MCT4 expression group were 40.7% and 43.4%, respectively, which were significantly lower than those in the low‐MCT4 expression group (72.0% and 61.9%, respectively). We further explored the prognostic value of MCT4 with different BCLC stages of patients with HCC. Of the 171 patients at stages 0–A, 75 were identified as having positive MCT4 expression in tumor cells. Patients with positive MCT4 expression in tumor cells had a poorer surgical prognosis than those with negative MCT4 expression in tumor cells (*P* = 0.033; Fig. 3C); however, similar results could not be verified in the 42 patients at stages B–C (*P* = 0.455; Fig. 3D). Moreover, high GLUT1 expression levels indicated poorer OS (*P* < 0.001; Fig. 3E) and higher TTR rates (*P* = 0.004; Fig. 3F). The 5‐year OS rates for the high‐ and low‐GLUT1 expression groups were 36.4% and 71.2%, respectively. These findings suggest that MCT4 and GLUT1 are important prognostic markers in patients with HCC. We evaluated whether MCT4 expression and GLUT1 expression were independently predictive of OS and TTR in patients with HCC using the multivariate Cox model. The results showed that MCT4 expression was an independent prognostic factor for OS in patients with HCC (hazard ratio [HR] = 1.617, 95% confidence interval [CI] = 1.102 ± 2.374, *P* = 0.014; Table 3).

### Prognostic significance of the metabolic index

**Figure 4:**
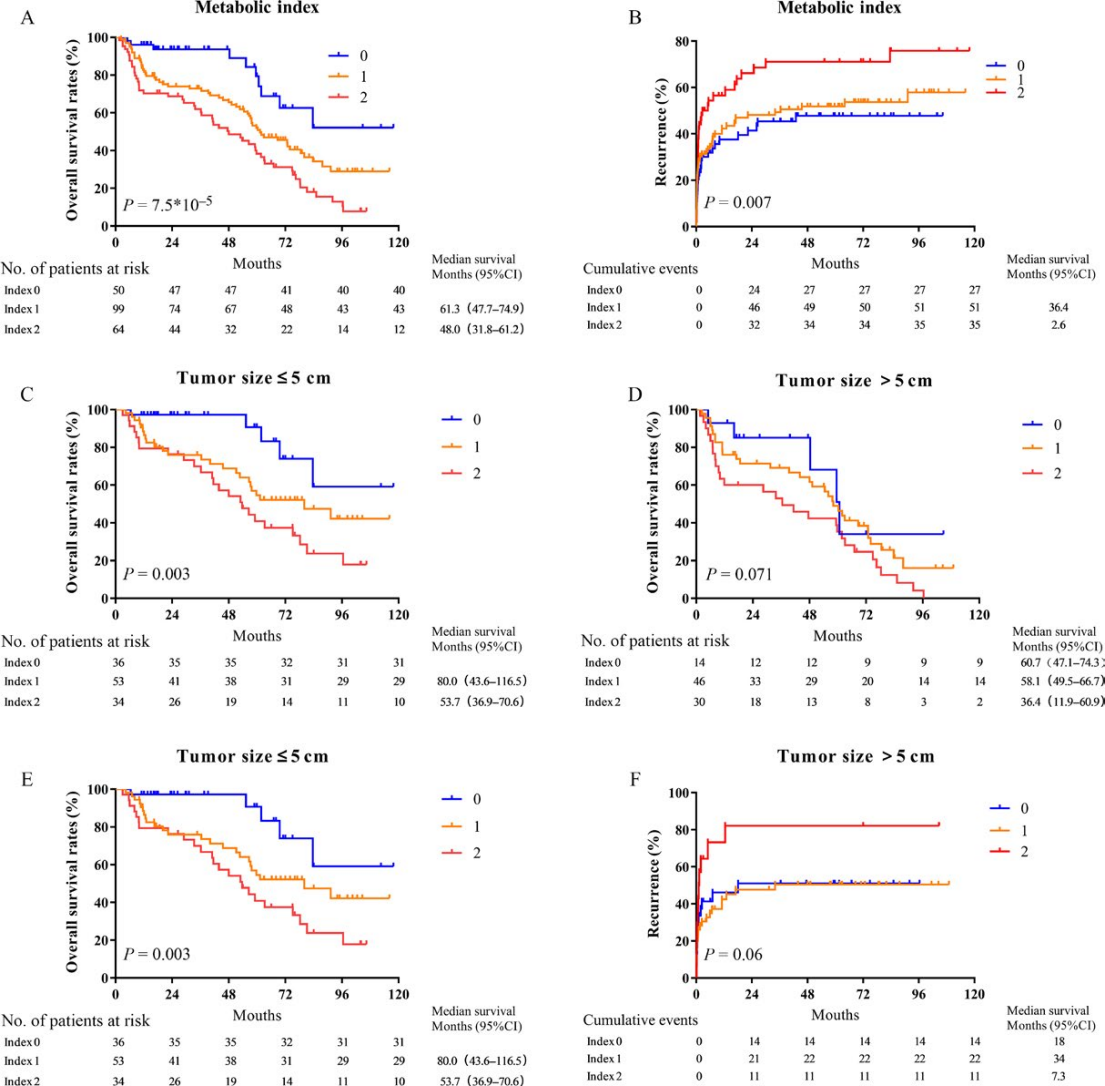
Prognostic significance of the metabolic index. (A, B) HCC patients with a high metabolic index have a poor OS (A) and short TTR (B) (*n* = 213). (C, D) Kaplan–Meier analysis of OS and TTR in patients according to tumor size, with 5 cm as the cutoff value. The OS ratio represents the prognostic significance of the metabolic index in the group with tumor size ≤5 cm (C) and the group with tumor size >5 cm (D). (E, F) The TTR ratio represents the prognostic significance of the metabolic index in the group with tumor size ≤5 cm (E) and the group with tumor size >5 cm (F).

The metabolism of cancer cells is very different from that of normal cells 21. Cancer cell energy is mainly supplied by anaerobic glycolysis, and the synthesis of fatty acids and glutamine is also higher than that in normal cells 22; in this process, glucose and lactate metabolism play a leading role 23. It is believed that the transmembrane transport of MCT4 via proton‐linked lactate plays a key role in maintaining glycolysis metabolism 24, whereas GLUT1 is one of the most important glucose transporters in tumor cells 25. Therefore, we incorporated GLUT1 and MCT4 expression status into the metabolic indices to assess the combined effects of GLUT1 and MCT4 on HCC. Low and high expression levels of MCT4 and GLUT1 were designated as 0 and 1, respectively. Subsequently, MCT4 and GLUT1 indices were added to obtain metabolic indices of 0–2. Patients were thus divided into three groups: 0, low MCT4 and low GLUT1 expression (*n* = 50); 1, low MCT4 and high GLUT1 expression or high MCT4 and low GLUT1 expression (*n* = 99); and 2, high GLUT1 and high MCT4 expression (*n* = 55). Kaplan–Meier survival analysis showed that patients with high metabolic indices had poor OS (*P* = 0.001; Fig. 4A) and TTR rates (*P* = 0.007; Fig. 4B). In addition, multivariate analysis showed that the metabolic parameters were independent negative prognostic factors for both OS (HR = 1.617, 95% CI = 1.102–2.374, *P* = 0.006) and TTR (HR = 1.348, 95% CI = 1.079 ± 1.685, *P* = 0.009) (Table 4). We also divided the patients into two subgroups according to tumor sizes. Patients with a tumor diameter >5 cm had a poorer prognosis than those with a tumor diameter ≤5 cm. Furthermore, the effect of the metabolic indices of the tumor cells on prognosis was examined in the two subgroups. The results showed that patients with positive metabolic index expression in tumor cells may have significantly shorter OS rates than those with negative metabolic index expression in tumor cells in the ≤5‐cm and >5‐cm subgroups (Fig. 4C–F). In general, the metabolic index is a potent prognostic factor for OS and TTR in patients with HCC.

**Table 4:** Univariate and multivariate analyses of prognostic factors in HCC

Variables	OS			TTR		
	Univariate *P* value	Hazard ratio (95% CI)	*P* value	Univariate *P* value	Hazard ratio (95% CI)	*P* value
Age (years)	0.795			0.301		
Gender	0.866			0.660		
HBsAg	0.277			0.934		
Serum AFP (ng/mL)	0.020		N.A.	0.061		
Tumor size (cm)	0.001	1.889 (1.120–3.186)	**0.017**	0.097		
Tumor number	0.000	1.691 (1.081–2.645)	**0.021**	0.028		N.A.
Microvascular invasion	0.001	2.021 (1.260–3.244)	**0.003**	0.000	2.410 (1.405–4.134)	**0.001**
Differentiation grade	0.569			0.406		
BCLC stage	0.007	1.542 (1.021–2.330)	**0.040**	0.892		
TNM stage	0.015		N.A.	0.011		N.A.
AST (μ/L)	0.815			0.929		
ALT (μ/L)	0.348			0.991		
Liver cirrhosis	0.372			0.287		
Metabolic index	0.001	1.617 (1.102–2.374)	**0.006**	0.007	1.348 (1.079–1.685)	**0.009**

OS, overall survival; TTR, the time to recurrence; HR, hazard ratio; CI, confidence interval; N.A., not applicable; MCT4, monocarboxylate transporter 4; GLUT1, glucose transporter 1.

Univariate analysis, Cox proportional hazards regression model. Multivariate analysis, Cox proportional hazards regression model. Variables were adopted by univariate analysis.

Bold values (*P* < 0.05) are statistically significant.

## Discussion

Abnormal glucose metabolism (glucose metabolism reprogramming) plays a key role in tumorigenesis and progression 26, and its regulatory mechanism has been a hot topic in the field of oncology. Cancer cell production of lactate, the primary end product of glycolysis, creates an acidic environment that favors tumor invasion and inhibits anticancer immune effects, thus supporting the growth and metastasis of cancer cells 10, 27. Previous studies report that MCT4, an efficient lactate exporter, promotes the hostile acidic microenvironment for glycolysis and tumor growth 28. In addition, a functional study showed that GLUT1, a key transporter in glucose metabolism, is crucial for HCC cell proliferation and migration 29. Recent studies have shown that MCT4 is highly expressed in patients with liver cancer and that high expression levels of MCT4 are a negative prognostic marker for OS and DFS 30. Therefore, whether MCT4 plays an important role in glycometabolism in hepatocellular carcinoma and its close relationship with GLUT1 is worth exploring. According to the BCLC staging system, patients in stages 0 and A are considered to be in the early stages of HCC and have a better outcome after radical resection. However, some of these early patients have poor prognosis in clinical practice. Our results suggest that the expression of MCT4 in tumor cells predicts worse survival in early patients (Fig 3C and D). Therefore, the findings of this study suggest that measuring the expression of MCT4 in tumor cells can discriminate poor prognosis in patients with early‐stage HCC. In the present study, we examined the expression of two important transporters, MCT4 and GLUT1, to determine their metabolic status in HCC and assess their prognostic value. Our data show that MCT4 and GLUT1 expression levels are significantly higher in HCC cells than in adjacent nontumorous hepatocytes. Due to the important functions of GLUT1 and MCT4, we found that patients with high MCT4/GLUT1 expression levels had shorter OS and TTR. In addition, the metabolic index that correlated with MCT4 and GLUT1 expression is an independent prognostic factor for OS and TTR in HCC.

Regarding the routine clinical and pathological features of HCC, the expression levels of MCT4 and GLUT1 are closely related to tumor size. MCT4 and GLUT1 are required for cell proliferation and tumor growth, and due to the high concentration of nutrients in cancer cells, GLUT1 and MCT4 may promote tumor microenvironment formation by regulating the concentration of nutrients 27. Furthermore, large tumors may promote the expression of MCT4 and GLUT1 29. Large tumors are usually associated with hypoxia and are known to induce HIF1α expression, which is associated with tumor size, and to promote GLUT1 and MCT4 expression in HCC 12. In addition, in chronically infected HBV and HCV livers, cirrhosis accompanies significantly reduced oxygen supply 31. Subsequent microenvironment‐induced HIF1α activates vascular endothelial growth factor and cyclooxygenase transcription to promote angiogenesis stabilization and activate matrix metalloproteinases 32. Consistent with these studies, we found that GLUT1 and MCT4 are highly expressed in tumors larger than 5 cm in diameter. In addition, hypoxia is associated with the upregulation of glycolysis 33. Lactate, a glycolysis product, triggers MCT4 expression through the activation of c‐Myc; this finding indicates that MCT4 is closely linked to glucose metabolism 34. In this study, we found a positive correlation between MCT4 expression and GLUT1 expression. These data suggest that there is a positive feedback loop between tumor growth and the upregulation of MCT4/GLUT1. This positive feedback loop may be important for HCC progression.

In conclusion, GLUT1 and MCT4 expression levels are significantly higher in tumors than in adjacent nontumorous hepatocytes, particularly in hepatoma tumors with diameters greater than 5 cm. Patients with high GLUT1 or MCT4 expression levels had a poor OS and short TTR, and the expression of MCT4 in tumor cells might discriminate poor prognosis in patients with early‐stage HCC. In addition, metabolic indices were independent prognostic factors for OS and TTR. Our study shows that the expression of GLUT1 and MCT4 determines the metabolic status of the tumor, and the combined expression of these two proteins may be a good prognostic biomarker and therapeutic target for patients with HCC.

## Conflict of Interest

None declared.
